# Predicting Pharmacological Treatment Response in Migraine Using AI/ML: A Scoping Review of the Evidence and Future Directions

**DOI:** 10.1002/phar.70085

**Published:** 2025-11-23

**Authors:** Martina Giacon, Salvatore Terrazzino

**Affiliations:** ^1^ Department of Pharmaceutical Sciences University of Piemonte Orientale Novara Italy

**Keywords:** artificial intelligence, CGRP monoclonal antibodies, machine learning, migraine, NSAIDs, OnabotulinumtoxinA, treatment response, Triptans

## Abstract

The treatment of migraine is hampered by inter‐individual variability, leading to an inefficient “trial and error” approach. Artificial intelligence (AI) and machine learning (ML) offer a path towards precision medicine by predicting therapeutic outcomes. This scoping review systematically evaluates the evidence for AI and ML models for predicting pharmacologic response in migraine. A systematic search of four databases (PubMed, Web of Knowledge, Cochrane Library, and OpenGrey) identified 12 eligible studies using AI/ML to predict acute or prophylactic response to migraine treatment. These studies, which date back to articles published in 2006 and have been increasingly published recently, used a wide range of methods, from classical algorithms like support vector machines to deep learning and probabilistic models. The models primarily utilized clinical phenotyping and neuroimaging data and reported high predictive accuracy for novel biologics (e.g., anti‐calcitonin gene‐related peptide monoclonal antibodies (CGRP mAbs)) and acute treatments (e.g., nonsteroidal anti‐inflammatory drugs (NSAIDs)). However, our systematic review finds that this apparent success is undermined by critical and pervasive methodological weaknesses. The central finding is that most studies relied solely on internal validation, carrying a high risk of overfitting, with external validation being exceptionally rare. Furthermore, several publications were based on overlapping patient cohorts, and a complete lack of biomarker or genetic data was noted. Consequently, the clinical application of AI and ML is currently stalled. Future progress depends on overcoming the “crisis of generalizability” by mandating external validation, addressing the “data bottleneck” with large, diverse datasets, and expanding data modalities to include “omic” data. These measures are critical to begin to realize the potential of AI and ML to personalize migraine treatment and significantly improve patient outcomes.

## 1 Introduction

Migraine is a major global health problem and the most common cause of disability in young women [1]. This debilitating neurological disorder is three to four times more common in women than in men, a difference influenced by hormonal, genetic, and structural factors [2]. The clinical picture involves recurrent episodes of moderate to severe headache with debilitating associated symptoms that are fundamental to the disease's pathophysiology [3]. For patients, this results in a profound burden, social stigmatization, and a significant gap between available treatments and individual needs [4]. In economic terms, the societal impact is equally large, with indirect costs from lost productivity accounting for the majority of the estimated annual burden of €173 billion in Europe [5].

A fundamental obstacle to the effective treatment of migraine is the great variability in patient response. This heterogeneity exists not only between different patients, but also within the same individual, where attack frequency, symptom severity, and triggers can vary considerably over time [6]. This variability within a patient, which is often underestimated in clinical practice, can lead to suboptimal therapeutic decisions. This challenge applies both to the selection of acute medication for individual migraine attacks and to the choice of prophylactic therapies for long‐term prevention. This includes the prescription of strong, expensive antimigraine drugs for mild headaches or, conversely, the exclusive use of simple analgesics for severe attacks, which can lead to unsatisfactory pain relief and potentially contribute to overuse of medication [6]. Consequently, despite advances in pharmacological treatment, a significant proportion of patients continue to receive inadequate treatment. Data from practice show that at least 56% of patients with migraine respond inadequately to acute treatment. This is a specific group of “inadequate responders” who suffer from greater disability and more comorbidities such as depression [7]. This therapeutic landscape is further complicated by the fact that even revolutionary treatments such as anti‐calcitonin gene‐related peptide (CGRP) therapies are only effective in around 50% to 60% of patients, highlighting the involvement of multiple pathophysiological pathways [6]. The current “trial and error” approach to migraine treatment is therefore often inefficient and costly.

To overcome this reactive “trial and error” approach, the burgeoning fields of artificial intelligence (AI) and machine learning (ML) offer a transformative way forward. These tools can analyze multimodal biomarker data to stratify patients and predict treatment efficacy, realizing the vision of precision medicine [8, 9]. This potential is underscored by models achieving near‐perfect diagnostic accuracy in the classification of migraine and its subtypes based on structural brain data [10, 11, 12], by the successful prediction of drug response in related neurological disorders such as epilepsy [13], and by a forward‐looking vision that integrates next‐generation technologies such as digital twins into headache treatment [14]. Despite this promising outlook, the path forward is often limited by significant methodological challenges, and the performance of these models does not inherently surpass that of well‐established regression methods. Nevertheless, these technologies are uniquely positioned to address the challenges posed by the variability of migraine. Their ability for data‐driven pattern analysis, sophisticated decision‐making, and accurate prediction provides a robust framework for personalizing treatment by efficiently processing large, complex, and heterogeneous data sets that exceed the analytical capabilities of humans [15, 16]. The overarching goal of integrating AI/ML into migraine treatment is to enable a paradigm shift from a reactive, empirical approach to a proactive, predictive approach: the “right drug for the right patient at the right time” [17].

As the application of AI/ML to predict response to migraine treatment is a highly dynamic and interdisciplinary field, with research scattered across neurology, informatics, and clinical informatics journals, a systematic review of the evidence is required to summarize the current evidence. A scoping review is the ideal method to capture this new landscape. Therefore, the main aim of this review is to systematically capture the existing research evidence on AI/ML models for predicting response to pharmacological treatment in migraine. The specific research questions guiding this review are:

1. What AI/ML architectures and algorithms have been used to predict response to pharmacologic treatment in migraine?
2. What data modalities (e.g., clinical, genomic, imaging, wearable data) have been used as input to these models?
3. For which classes of acute and prophylactic medications were predictive models developed?
4. How were the performance and generalizability of these models evaluated?
5. What are the main research gaps and challenges identified in the literature?

## 2 Methods

### 2.1 Study Design and Protocol

This scoping review was designed and conducted in accordance with the methodological framework for scoping reviews proposed by Arksey and O'Malley and is reported according to the Preferred Reporting Items for Systematic reviews and Meta‐Analyses extension for Scoping Reviews (PRISMA‐ScR) checklist [18] (Table S1). A scoping review method was preferred to a systematic review in order to comprehensively capture the scope, nature, and characteristics of this broad and heterogeneous field of research. Given the variability in study designs and outcome definitions, a quantitative synthesis such as a meta‐analysis was considered premature. The protocol was defined in advance of the literature search and registered on the International Platform of Registered Systematic Review and Meta‐analysis Protocols (INPLASY) (registration number 202560067. doi: https://doi.org/10.37766/inplasy2025.6.0067).

### 2.2 Eligibility Criteria

The inclusion and exclusion criteria were defined based on the *Population*, *Concept*, and *Context* framework. Studies were included if they focused on a *Population* of patients of any age with a diagnosis of any migraine subtype. The core *Concept* was the application of any AI/ML model (e.g., supervised, unsupervised, or deep learning algorithms) with the explicit aim of predicting a patient's response to a specific pharmacological treatment. All research *Contexts* were considered, including clinical trials, retrospective and prospective cohort studies, and analyses of real‐world data. Reviews, editorials, commentaries, case reports, and conference abstracts were excluded, as were studies in animal models or those focusing solely on diagnosis, prediction of seizures, or non‐pharmacological interventions. No language restrictions were applied.

### 2.3 Information Sources and Search Strategy

A comprehensive literature search was conducted in four electronic databases (PubMed, Web of Knowledge, Cochrane Library, and OpenGrey), from the start of the database to July 4, 2025. The search strategy was developed by combining MeSH terms and keywords for three core concepts: (i) migraine and headache disorders, (ii) artificial intelligence and machine learning, and (iii) treatment outcomes and drug therapy. The search was conducted using a structured query combining MeSH terms and free text keywords. The foundational search strategy was formulated for PubMed and subsequently modified for the other selected databases (Web of Science, Cochrane Library, and OpenGrey) to align with their specific syntax:

(“migraine disorders”[MeSH] OR migraine[tiab]) AND (“artificial intelligence”[MeSH] OR “machine learning”[MeSH] OR “deep learning”[tiab] OR “neural networks, computer”[MeSH] OR “artificial intelligence”[tiab] OR “machine learning”[tiab] OR ai[tiab] OR ml[tiab] OR dl[tiab] OR “neural network*”[tiab] OR “intelligent system*”[tiab] OR “computational intelligence”[tiab] OR “predictive modeling”[tiab]) AND (“drug therapy”[MeSH] OR “treatment outcome”[MeSH] OR treatment*[tiab] OR therap*[tiab] OR medication*[tiab] OR drug*[tiab] OR pharmacotherap*[tiab] OR “pharmacological treatment”[tiab] OR “drug response”[tiab]).

### 2.4 Study Selection

A comprehensive list of the search strings applied to each database can be found in Table S2. The selection procedure was carried out in two successive stages. First, two reviewers (M.G. and S.T.) independently checked the titles and abstracts of all identified records against the predefined eligibility criteria. In a second step, the full texts of all articles identified as potentially relevant in the first phase were retrieved and reviewed by the same two independent reviewers for final inclusion in the scoping review. Any discrepancies or disagreements in both phases of the screening process were resolved in a formal discussion to reach consensus.

### 2.5 Data Extraction

Two reviewers (M.G. and S.T.) independently extracted data from the included studies using a data collection form developed in Microsoft Excel. Variables extracted included: study identifiers and design, population characteristics, pharmacologic class and treatment response definition, the full AI/ML pipeline (including algorithms, feature selection, and validation strategy), and key outcomes, such as the primary metrics of the best model (e.g., Area Under the Receiver Operating Characteristic Curve [AUC]) and its key predictive features.

### 2.6 Data Synthesis and Analysis

The evidence was analyzed in two main phases. First, a descriptive numerical analysis was performed to summarize the characteristics of the included studies. This involved calculating frequencies and distributions for variables such as year of publication, geographical location, study design, and sample size. Second, a thematic synthesis was performed to address the specific objectives of the study. The extracted data were therefore thematically organized according to the following core concepts:

- AI/ML methods: grouping the studies according to the type of algorithm used (e.g., tree‐based models, deep learning, support vector machines (SVMs)).
- Data modalities: categorization of studies based on the input data used (e.g., clinical, imaging, genomic, portable data).
- Pharmacologic classes: grouping studies according to the specific acute or prophylactic drug classes for which they attempted to predict a response.

This dual approach provides a comprehensive mapping of the research landscape and identifies important trends, common practices, and significant gaps in the existing literature.

## 3 Results

### 3.1 Study Selection and Characteristics

**FIGURE 1:**
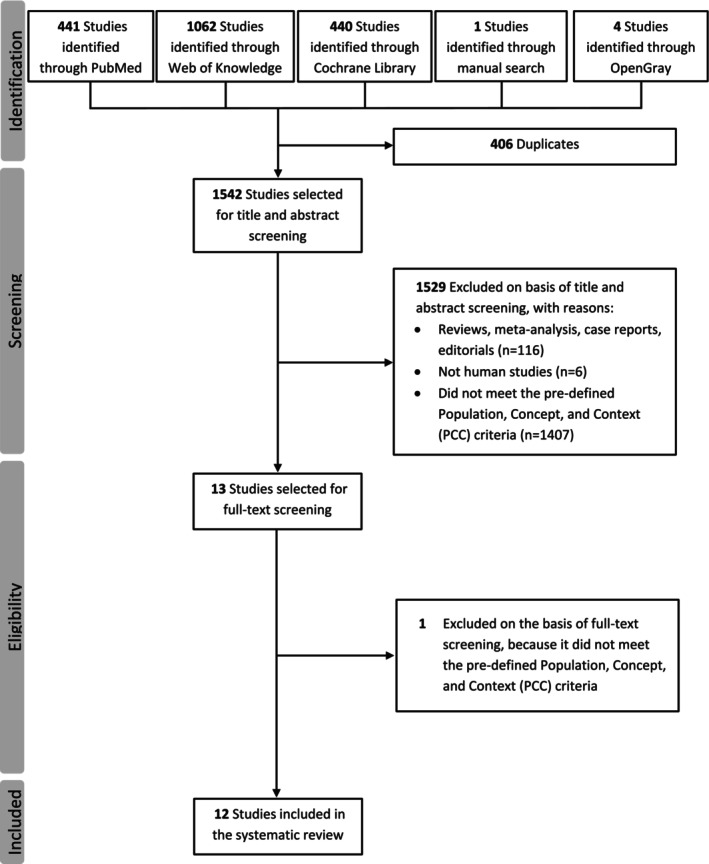
PRISMA flow diagram of the study selection process. The diagram illustrates the flow of information through the different phases of the review, including the number of records identified through database searching, records screened after duplicate removal, full‐text articles assessed for eligibility, and the final number of studies included in this scoping review.

The initial literature search in PubMed, Web of Knowledge, Cochrane Library, OpenGray and the manual search yielded a total of 1948 entries. After removing 406 duplicate entries, 1542 unique studies were transferred to the title and abstract screening phase. In this phase, a large majority of articles (*n* = 1529) were excluded. Apart from ineligible article types (e.g., reviews, editorials; *n* = 116) and studies not conducted in humans (*n* = 6), the largest group of exclusions (*n* = 1407) consisted of studies that did not meet our specific Population, Concept, and Context criteria when their title and abstract were reviewed. Our comprehensive search query was intentionally broad to minimize the risk of missing relevant studies, which resulted in the identification of many articles clearly outside the scope of our review. These exclusions encompassed a wide range of articles. For example: (a) Violation of the “Concept”: a substantial number of studies examined predictors of treatment response in migraine but did not use an AI/ML model, relying instead on traditional statistical methods. (b) Violation of the “Population”: another large group consisted of studies that did not address migraine at all. (c) Violation of the “Concept”: a smaller proportion of excluded articles applied AI/ML, but for purposes other than predicting response to pharmacologic treatment, such as diagnosis, classification, or predicting response to nonpharmacologic interventions (e.g., neurostimulation, acupuncture). Following this extensive title and abstract screening, the remaining 13 studies were screened for eligibility by a full‐text review. Of these studies, one was excluded because the reported results did not meet the predefined criteria of the review. This process resulted in a final selection of 12 studies that met all inclusion criteria for this systematic review [19, 20, 21, 22, 23, 24, 25, 26, 27, 28, 29, 30]. A detailed diagram of this selection process can be found in Figure 1. A summary of the design and population characteristics can be found in Table 1, and a summary of the methodology and performance of the AI/ML models of the included studies can be found in Table 2 and Table S3.

**TABLE 1:** Summary of included studies—design and population characteristics.

First author (year) [ref]	Location	Design	Total sample size (R, NR)	Migraine subtype(s)	Age, years (mean)	Sex (% *F*)	Drug(s) evaluated	Definition of treatment response/response assessment methodology
Maas (2006) [19]	The Netherlands/UK	randomized, placebo‐controlled trial	638 (N/A, N/A)	NS	39	89%	Oral Sumatriptan (25 mg, 50 mg, and 100 mg doses) and Placebo	The study modeled the time course of response using a Hidden Markov Model with three states (no relief, relief, pain‐free), which were linked to observed patient‐reported headache scores on a 4‐point scale (0 = no pain, 1 = mild, 2 = moderate, 3 = severe)
Parrales Bravo (2019) [20]	Spain	Retrospective	173 (NS, NS)	CM	NS	NS	Onabotulinum toxinA	For a small subgroup: as a > 30% reduction in the HIT‐6 score. For the main model: As a ‘high’ or ‘low’ outcome based on a composite score derived from both a 4‐point scale for migraine day reduction and a 4‐point scale for adverse effects
Lu (2022) [21]	China	Prospective	610 (326, 284)	NS	35.1 (R), 35.9 (NR)	79.0%	NSAIDs (aspirin, ibuprofen, naproxen, celecoxib) acetaminophen	≥ 50% reduction in headache intensity (VAS score) at 2 h post‐dose, with reproducibility and no headache recurrence within 24 h
Wei (2022) [22]	China	Cross‐sectional	70 (35, 35)	MwoA	33.9 (R), 35.7 (NR)	90.0%	NSAIDs	≥ 50% reduction in pain intensity (VAS score) 2 h after taking medication
Wei (2022) [23]	China	Prospective, cross‐sectional	70 (35, 35)	MwoA	33.9 (R), 35.7 (NR)	90.0%	NSAIDs (including ibuprofen and aspirin)	≥ 50% reduction in pain intensity (VAS score) 2 h after taking medication, occurring on at least two occasions
Gonzalez‐Martinez (2022) [24]	Spain	Prospective	712 (N/A, N/A)	CM (83.8%) EM (18.7%)	48.5	93.3%	Anti‐CGRP mAbs (erenumab, galcanezumab, fremanezumab)	Multi‐class outcome: ≥ 30%, ≥ 50%, and ≥ 75% reduction in MHD at 6, 9, and 12 months
Martinelli (2023) [25]	Italy	Retrospective	145 (35, 38)^a^	CM and HFEM	NS	83.4% (121 out of 145)	Onabotulinum toxinA	≥ 50% reduction in monthly migraine days (MMD) after the 4th treatment cycle
Wei (2023) [26]	China	Prospective, cross‐sectional	111 (62, 49)	NS for the entire cohort, but the presence of aura was a recorded variable	31.5 for the training set and 31.0 (median) for the testing set	77.7% (training set), 91.3% (testing set)	NSAIDs (including ibuprofen, aspirin, naproxen, celecoxib) acetaminophen	A composite definition based on a headache diary over 3 months, requiring: (1) no pain after 2 h; OR (2) pain improvement from moderate/severe to mild/none (or ≥ 50% VAS decrease) after 2 h; AND (3) the effect being repeatable in at least 2 of 3 attacks; AND (4) no recurrence or need for rescue medication within 24 h
Wei (2024) [27]	China	Cross‐sectional	64 (32, 32)	MwoA	33.2 (R), 34.9 (NR)	90.6%	NSAIDs	≥ 50% reduction in headache intensity (VAS score) at 2 h post‐dose, with reproducibility and no headache recurrence within 24 h.
Romozzi (2024) [28]	Italy	Prospective	336 (N/A, N/A)	CM (89.9%) EM (11.1%)	48.2	80.1%	Anti‐CGRP mAbs (erenumab, galcanezumab, fremanezumab)	Multi‐class outcome: response was categorized into four levels of MHD reduction: < 25%, 25%–50%, 50%–75%, and > 75%
Wei (2024) [29]	China	Prospective	118 (59, 59)	EM	Median 35.0 (R) 33.0 (NR)	83.1%	NSAIDs	Composite criteria: ≥ 50% VAS reduction or pain‐free at 2 h, no recurrence within 24 h, and effect in ≥ 2 of 3 attacks
Chiang (2024) [30]	USA	Prospective	4260^b^	CM and EM	42.8	78.8%	7 Preventive Classes^c^	≥ 30% MHD reduction

Abbreviations: CGRP mAbs, calcitonin gene‐related peptide (CGRP) monoclonal antibodies; CM, chronic migraine; EM, episodic migraine; HFEM, high‐frequency episodic migraine; HIT‐6, headache impact test 6‐item; MHD, monthly headache days; MMD, mean monthly migraine days; MwoA, migraine without aura; NR, non‐responders; NS, not stated; *NSAID*s, nonsteroidal anti‐inflammatory drugs; R, responders; RCT, randomized, placebo‐controlled trial; Ref, reference; VAS, visual analog scale.

^a^ The machine learning models were specifically trained on a subset comparing 35 excellent/good responders (R) against 38 non‐responders (NR).

^b^ Multi‐drug study with responders/non‐responders calculated separately for each of the seven drug classes.

^c^ CGRP mAbs, onabotulinumtoxinA, beta‐blockers, tricyclic antidepressants, topiramate, verapamil, gabapentin.

**TABLE 2:** Methodological and performance summary of AI/ML models.

First author (year) [ref]	AI/ML used	Performance metrics	Key predictive features	Best AI/ML algorithm	Best performance/key model outcome(s)	Validation
Maas (2006) [19]	HMM	Parameter estimation (EC_50_); Visual predictive checks	Simulated sumatriptan concentration; Headache scores	HMM	Modeled time course of response; EC_50_ estimation (9 ng/mL)	Not performed (performance based on goodness‐of‐fit to the full dataset)
Parrales Bravo (2019) [20]	Multiple classifiers (e.g., NB, k‐NN, C4.5, RF, SVM), Clustering (k‐means, EM), SA optimization	Acc, Sens, Spec	1st Infilt: GPT, prior drug trials, chronicity. 2nd Infilt: GON response, retroocular component	Primary task (response prediction): Farthest‐First + SA Secondary task (HIT‐6): C4.5 and NBTree	Acc: 88.5% (1st Infilt); 85.9% (2nd Infilt)	Internal (10‐fold CV; LOOCV on HIT‐6 subset)
Lu (2022) [21]	LR, SVM, DT, MLP	AUC, Sens, Spec	Disease duration, intensity (VAS), frequency, GAD‐7, PHQ‐9, PSQI scores	SVM (all features)	AUC: 0.744 (CI: 0.657–0.818)	Internal (80/20 train/test split; 10‐fold CV on train)
Wei (2022) [22]	MLR, SVM	AUC, Acc, Sens, Spec	rs‐fMRI FC between left amygdala and ipsilateral CAL/CAU	SVM	AUC: 0.896 Acc: > 89%	Internal (80/20 train/test split; 5‐fold CV on train)
Wei (2022) [23]	SVM	AUC, Sens, Spec	Abnormal FNC patterns between brain networks	SVM	AUC: 0.93	Internal (75/25 train/test split; 5‐fold CV)
Gonzalez‐Martinez (2022) [24]	RF with SFS and BSO	Acc, AUC, F1, PPV, TPR	Clinical response at earlier time points; specifically, HDM at 3 and 6 months	RF	AUC: 0.87–0.98 F1: 0.70–0.97	Internal (80/20 train/test split)
Martinelli (2023) [25]	RF, SVM, ANN, ANFIS; FCM clustering; ReliefF selection; SMOTE	Acc, Sens, Spec, Precision, F‐measure, AUC	HFEM: Age at onset, opioid use, HADS‐a, MIDAS CM: No small set of features found	RF (on HFEM subgroup)	AUC: 0.909^a^ Acc: 85.7%	Internal (Balanced 10‐fold Monte Carlo CV)
Wei (2023) [26]	Deep Learning (3D CNNs)	Accuracy, AUC, Recall, Precision, F1‐score, PLR, NLR, Cutoff	Raw 3D T1‐weighted MRI images	3D ResNet18	AUC: 0.82; Accuracy: 0.78	Internal (80/20 train/test split)
Wei (2024) [27]	SVM with GCA	AUC, Sens, Spec	rs‐fMRI causal connectivity between ACC and LG	SVM (bidirectional)	AUC: 0.939	Internal (80/20 train/test split; 10‐fold CV on train)
Romozzi (2024) [28]	RF (chosen over GBM, LR, DT); SBFS; Grid Search CV	Acc, Precision, Recall, F1, AUC‐ROC	Reduction in MHD from prior time point; years since chronicization, HIT‐6, acute medication use	RF	Internal: AUC 0.76 External: AUC 0.78	Internal and External (80/20 train/test split; validated on separate external cohort)
Wei (2024) [29]	Multiple classifiers (e.g., RF, LR, SVM, KNN, XGBoost); LASSO, RFE selection	ROCAUC, PRAUC, BACC, Sens, F1	Functional: Left insula, left transverse temporal gyrus. Structural (GMV): Right sup. frontal gyrus, bilateral postcentral gyrus, left precuneus	RF	Internal: ROCAUC 0.711 External: ROCAUC 0.631	Internal and External (70/30 train/test split; validated on public external dataset)
Chiang (2024) [30]	AutoML (GBM, DRF, GLM, XGBoost, Stacked Ensembles) with TabNet and SHAP	AUC, Acc, Precision, Recall, F1	Baseline MHD, Age, BMI, attack duration, prior trial responses, specific migraine features & triggers	GBM (for CGRP mAbs)	AUC: 0.825 Acc: 0.80	Internal (85/15 train/test split from large single‐center dataset)

Abbreviations: Acc, accuracy; ACC, anterior cingulate cortex; AI/ML, artificial intelligence/machine learning; ANFIS, adaptive neuro‐fuzzy inference system; ANN, artificial neural network; AUC, area under the (receiver operating characteristic) curve; AutoML, automated machine learning; BACC, balanced accuracy; BSO, bayesian search optimization; C4.5, a specific decision tree algorithm; CAL, calcarine sulcus; CAU, caudate nucleus; CI, confidence interval; CM, chronic migraine; CV, cross‐validation; DRF, distributed random forest; DT, decision tree; EM, expectation–maximization; FC, functional connectivity; FCM, Fuzzy C‐means clustering; FNC, functional network connectivity; GAD‐7, generalized anxiety disorder 7‐item scale; GBM, gradient boosting machine; GCA, granger causality analysis; GLM, generalized linear model; GMV, gray matter volume; GON, greater occipital nerve; GPT, glutamic pyruvic transaminase; HADS‐a, hospital anxiety and depression scale—anxiety subscale; HDM, headache days per month; HFEM, high‐frequency episodic migraine; HMM, hidden markov model; HIT‐6, headache impact test 6‐item; k‐NN, k‐nearest neighbors; LASSO, least absolute shrinkage and selection operator; LG, lingual gyrus; LOOCV, leave‐one‐out cross‐validation; LR, logistic regression; MHD, monthly headache days; MIDAS, Migraine Disability Assessment Score; MLP, multilayer perceptron; MLR, multivariable logistic regression; NB, naive bayes; PHQ‐9, patient health questionnaire 9‐item; PPV, positive predictive value; PRAUC, area under the precision‐recall curve; PSQI, Pittsburgh Sleep Quality Index; Ref, reference; ReliefF, A feature selection algorithm; RF, random forest; RFE, recursive feature elimination; ROCAUC, area under the receiver operating characteristic curve (synonymous with AUC); rs‐fMRI, resting‐state functional magnetic resonance imaging; RT, random tree; SA, simulated annealing; SBFS, sequential backward feature selection; Sens, sensitivity; SFS, sequential forward selector; SHAP, SHapley Additive exPlanations; SMOTE, synthetic minority oversampling technique; Spec, specificity; SVM, support vector machine; TabNet, A deep learning model for tabular data; TPR, true positive rate (sensitivity); VAS, visual analog scale.

^a^ The reported predictive performance was achieved only in the subgroup of 32 patients with high‐frequency episodic migraine (HFEM), while the models failed to perform in the chronic migraine (CM) cohort.

The 12 eligible studies show that although the literature on ML for predicting drug response in migraine has grown rapidly since 2019 [20], the foundational work dates back to 2006 [19]. This has been followed by an acceleration in publications, with four studies [21, 22, 23, 24] in 2022, two [25, 26] in 2023 and four [27, 28, 29, 30] in 2024. The vast majority of studies (11 out of 12) are from centers in Europe (Spain, Italy, Netherlands/United Kingdom) and East Asia (China), with only one study from North America (United States), indicating globally distributed but focused research.

Sample sizes vary by more than two orders of magnitude, from neuroimaging studies [22, 23, 26, 27, 29] with only 64 to 70 participants to a large‐scale registry analysis with 4260 patients [30]. The median cohort size in the included studies is 159 patients, suggesting that most studies are based on single center or regional datasets rather than large, multinational consortia.

The most commonly modeled class of acute medications is non‐steroidal anti‐inflammatory drugs (NSAIDs), which have been the subject of six different studies [21, 22, 23, 26, 27, 29], often using neuroimaging data with ML or deep learning. The triptan class is also represented, with an early study from 2006 modeling the response to sumatriptan [19]. Among preventive agents, the most commonly modeled drug classes are onabotulinumtoxinA [20, 25, 30] and anti‐CGRP monoclonal antibodies [24, 28, 30], each appearing in three studies. Based on the included literature, no ML studies have been published on small molecule CGRP receptor antagonists (gepants) or serotonin 5‐HT1F receptor agonists (ditans).

### 3.2 AI/ML Methods: A Landscape Defined by Three Methodological Approaches

The AI/ML methods used can be divided into four approaches (Table 3), whereby the landscape is dominated by classical models. This is also reflected in the choice of algorithms in the literature reviewed: support vector machines (SVMs) were the most commonly used algorithm and appeared in seven studies [20, 21, 22, 23, 25, 27, 29], closely followed by tree‐based models (e.g., random forest), which were used in six studies [20, 24, 25, 28, 29, 30].

**TABLE 3:** Classification of Studies by AI/ML Methodology.

Algorithm group	Description of the approach	Studies in this group
1. Probabilistic Time‐Course Modeling	This approach uses probabilistic models like Hidden Markov Models (HMM) to describe the dynamic progression of a disease through different states over time, rather than performing a simple binary classification.	Maas (2006) [19]
2. Broad Exploration of Classical Models	These studies tested a wide and diverse range of well‐established machine learning algorithms. The primary goal was a large‐scale comparison to identify the best‐performing model from a pool of different classifier families (e.g., linear, tree‐based, instance‐based, probabilistic).	Parrales Bravo (2019) [20] Lu (2022) [21] Wei (2022) [22] Wei (2022) [23] Wei (2024) [27] Wei (2024) [29]
3. Focused Ensemble Models (Tree‐Based)	This group of studies did not test a wide variety of algorithms. Instead, they selected a single tree‐based ensemble method (specifically, Random Forest) as their primary tool. Their research efforts were concentrated on optimizing this specific model through feature selection and hyperparameter tuning.	Gonzalez‐Martinez (2022) [24] Romozzi (2024) [28]
4. Deep learning or other neural‐network‐based architectures	These studies employed state‐of‐the‐art methods that go beyond the classical set. This group is defined by the use of Artificial Neural Networks (ANN), Deep Learning architectures (like 3D Convolutional Neural Networks (CNNs) or TabNet), or Automated Machine Learning (AutoML) pipelines to build and optimize their predictive models.	Martinelli (2023) [25] Wei (2023) [26] Chiang (2024) [30]

An independent, early approach, referred to as *probabilistic time‐course modeling*, is represented by the foundational work of Maas et al. [19]. This study used a Hidden Markov Model (HMM), a probabilistic framework developed not for binary classification but for modeling the dynamic progression of a migraine attack through different states (e.g., no relief, relief, pain‐free) over time, incorporating pharmacokinetic data.

The most widely used strategy, categorized as *broad exploration of classical models*, was used in six of the twelve studies. This approach tests a broad and diverse range of established ML algorithms. The primary goal is a large‐scale comparison to determine the best performing model from a pool of different classifier families (e.g., linear, tree‐based, instance‐based, probabilistic). This includes the work of Parrales Bravo et al. [20], who evaluated an extensive list of classifiers, and the studies of Lu et al. [21] and Wei et al. [22, 23, 27, 29], who often used basic models such as SVMs and logistic regression.

A third approach, referred to as *focused ensemble models* (tree‐based), was identified in two studies. In contrast to the broad exploratory group, these studies did not test a variety of algorithms. Instead, as seen in the work of Gonzalez‐Martinez et al. [24] and Romozzi et al. [28], they chose a single tree‐based ensemble method—namely Random Forest—as their primary tool. Their research efforts then focused on optimizing this particular model through rigorous feature selection and hyperparameter tuning.

Finally, a fourth group of studies is characterized by the use of *deep learning or other neural‐network‐based architectures* [25, 26, 30]. This group is characterized by the use of methods that go beyond the classical set. The study by Martinelli et al. [25] investigated artificial neural networks (ANN) and adaptive neuro‐fuzzy inference systems (ANFIS). At the same time, Wei et al. [26] represented a significant advance by being the first to apply a 3D Convolutional Neural Network (CNN), specifically a ResNet18 architecture, directly to raw structural Magnetic Resonance Imaging (MRI) images to predict response to treatment without prior feature engineering. Building on these advances, the recent study by Chiang et al. [30] is the first to employ a deep learning architecture for tabular data (TabNet) and use an automated ML (AutoML) pipeline to build and optimize their final predictive models from clinical registry data.

### 3.3 Data Modalities: The Primacy of Rich Phenotyping and Neuroimaging

The ML algorithms in the included studies were applied to three primary data modalities, as listed in Table 4: clinical phenotyping, neuroimaging, and a combination of pharmacokinetic and clinical data. The patterns of performance clearly reflect the data modality and dimensionality of the underlying characteristics for each category.

**TABLE 4:** Classification of studies by input data modality.

Data modality group	Description of input data	Studies in this group
1. Clinical phenotyping	These studies used structured data collected directly from patients or their clinical records. This includes demographics, patient‐reported history (e.g., disease duration, medication use), clinical examination findings, and scores from standardized scales (e.g., HIT‐6, MIDAS, GAD‐7).	Parrales Bravo (2019) [20] Lu (2022) [21] Gonzalez‐Martinez (2022) [24] Martinelli (2023) [25] Romozzi (2024) [28] Chiang (2024) [30]
2. Pharmacokinetic & clinical data	This study combined patient‐reported clinical data (headache scores) with simulated population pharmacokinetic data (drug plasma concentrations) to model the concentration‐effect relationship over time.	Maas (2006) [19]
3. Neuroimaging	These studies used high‐dimensional data derived from brain imaging as their primary predictive features. The input features were typically not raw clinical variables but rather engineered metrics from fMRI or MRI, such as functional connectivity, causal connectivity, percentage of amplitude oscillations, and gray matter volume. One study used raw structural images as direct input for a deep learning model.	Wei (2022) [22] Wei (2022) [23] Wei (2023) [26] Wei (2024) [27] Wei (2024) [29]

Abbreviations: fMRI, functional magnetic resonance imaging; GAD‐7, generalized anxiety disorder 7‐item scale; HIT‐6, headache impact test 6‐item; MIDAS, Migraine Disability Assessment Score; MRI, magnetic resonance imaging.

#### 3.3.1 Clinical Phenotyping

The majority of studies relied on clinical phenotyping, with six of the twelve studies using this modality exclusively. These studies created models based on structured data collected directly from patients, including demographic data, patient‐reported medical history, clinical examination findings, and scores from standardized scales such as Headache Impact Test 6‐item (HIT‐6) and Migraine Disability Assessment Score (MIDAS) [20, 21, 24, 25, 28, 30]. The performance of these models was highly variable, with reported AUCs in some studies as high as 0.98 [24], while others were lower (e.g., 0.744) [21].

#### 3.3.2 Pharmacokinetic and Clinical Data

A unique, early approach was taken by Maas et al. [19], who developed a model based on the combination of clinical data (headache scores) and simulated population pharmacokinetic data (sumatriptan plasma concentrations). This study is characterized by its focus on modeling the concentration–response relationship over time.

#### 3.3.3 Neuroimaging

As shown in Table 4, five of the twelve studies used neuroimaging data as the primary input [22, 23, 26, 27, 29]. These models were not fed with raw clinical variables, but with high‐dimensional constructed metrics from fMRI or MRI, such as functional connectivity (FC), causal connectivity, percentage of amplitude oscillations (PerAF), and gray matter volume (GMV). The study by Wei et al. [26] is an exception as it applies a deep learning model directly to raw T1‐weighted 3D structural images. These structural and functional MRI signatures, when integrated into either classical algorithms or more recent deep learning models, have shown high reported predictive performance. For example, the study by Wei et al. [27], which used causal connectivity metrics with an SVM, achieved an AUC of 0.939 in a cohort of 64 patients. A similar pipeline by Wei et al. [22], which used functional connectivity in 70 patients, achieved an AUC of 0.896, and the work by Wei et al. [23], which used a different functional connectivity approach, achieved an AUC of 0.891. The study by Wei et al. [29] combined functional (PerAF) and structural (GMV) metrics and achieved an Area Under the Receiver Operating Characteristic Curve (ROCAUC) of 0.711.

### 3.4 Reported Predictive Performance by Pharmacological Class and Therapeutic Goal

This section directly addresses research question 3 (*For which classes of acute and prophylactic medications were predictive models developed*?) by systematically summarizing the reported predictive performance of AI/ML models for the specific classes of acute and prophylactic medications identified in the literature.

#### 3.4.1 Models for Predicting Response to Preventive (Prophylactic) Therapies

The aim of these models is to predict a patient's long‐term response, which is usually measured as a reduction in monthly migraine or headache days over several months.

##### 3.4.1.1 Prophylactic Monoclonal Antibodies (CGRP‐mAbs)

**FIGURE 2:**
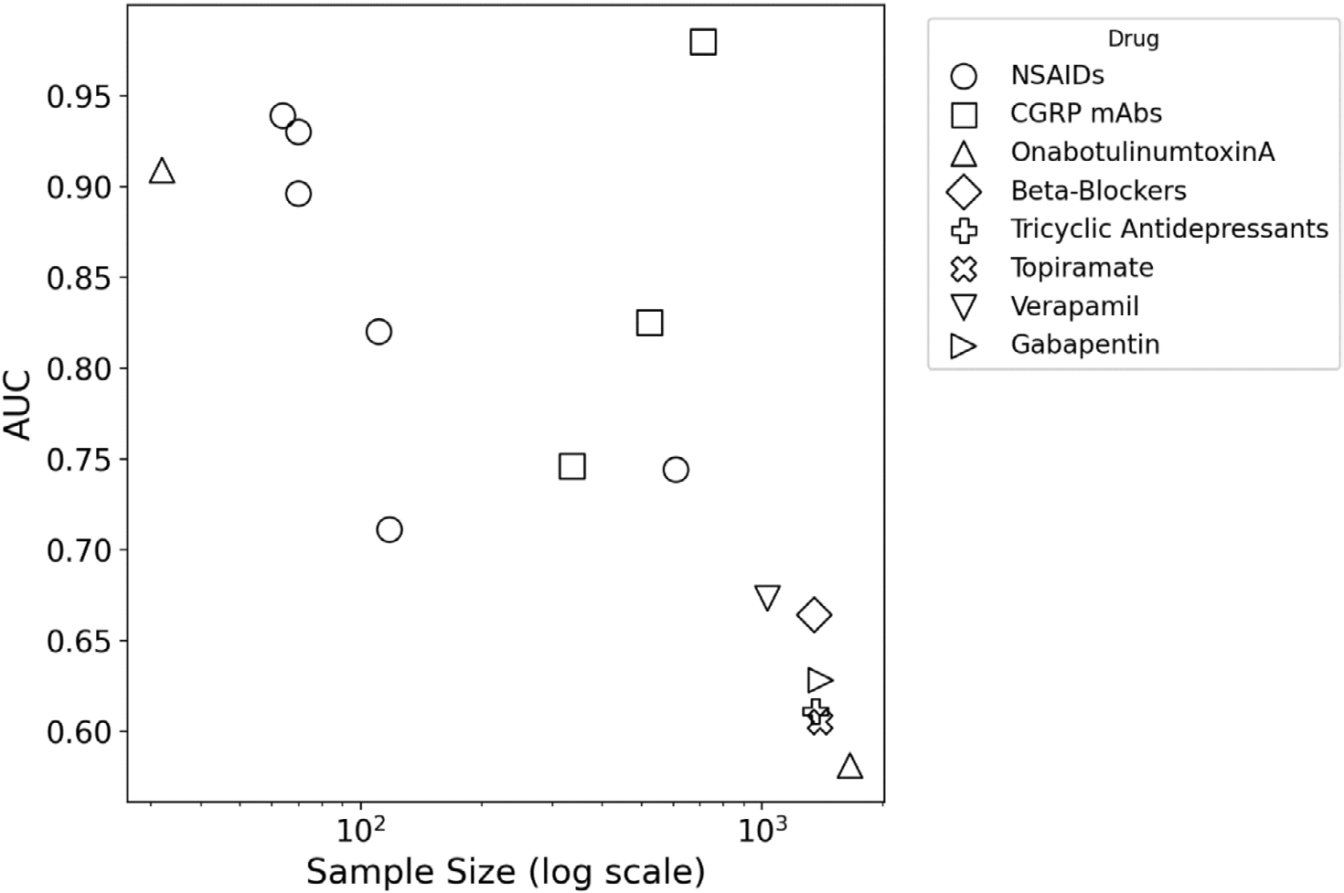
Predictive Performance (Area Under the Curve (AUC)) vs. Cohort Size for Artificial Intelligence (AI)/Machine Learning (ML) Models Across Different Pharmacological Classes. The scatter plot visualizes the relationship between the predictive performance of AI/ML models, measured by the AUC on the y‐axis, and the cohort size (Sample Size, on a logarithmic scale) on the x‐axis. CGRP mAbs: Calcitonin gene‐related peptide monoclonal antibodies; NSAIDs: Nonsteroidal anti‐inflammatory drugs.

Three studies, listed in Figure 2, have investigated the efficacy of CGRP‐mAbs. Reported performances vary, with Gonzalez‐Martinez et al. [24] reporting an AUC of 0.980 using extensive clinical and patient‐reported data. Chiang et al. [30] achieved an AUC of 0.825 using a gradient‐boosting machine, and Romozzi et al. [28] achieved an AUC of 0.746.

##### 3.4.1.2 OnabotulinumtoxinA

Three studies focused on botulinum toxin. The study by Martinelli et al. [25] powerfully illustrates the risk of overfitting and poor generalizability. The authors reported a model with a high AUC of 0.909, but this result was limited to a very small and specific subgroup of 32 patients with high‐frequency episodic migraine. Critically, this apparent success was contrasted by the model's complete failure when applied to the larger chronic migraine cohort within the same study, leading the original authors to conclude that their model could not accurately predict response [25]. This example serves not as a showcase of a successful model, but as a cautionary tale that directly supports our review's central finding regarding the crisis of generalizability. In stark contrast, the study by Chiang et al. [30] with multiple drugs yielded an AUC of 0.581 for this drug class in a much larger cohort. A third study by Parrales Bravo et al. [20] also examined this class of drugs, but reported accuracy (~85%) rather than AUC as the primary performance measure.

##### 3.4.1.3 Classical Oral Preventive Agents

Chiang et al. [30] provided the first evidence of ML for several known oral agents. In contrast to the performance of their CGRP model, the reported AUCs for these drugs were lower, ranging from 0.605 (topiramate) to 0.673 (verapamil).

#### 3.4.2 Models for Predicting Response to Acute (Abortive) Therapies

The goal of these models is to predict a patient's response to a single dose of medication for an active migraine attack, typically measured by pain reduction or freedom at 2 h post‐dose.

##### 3.4.2.1 NSAIDs

The analysis of the acute response to NSAIDs was the subject of six studies.^21–23.26,27,29^ It is noteworthy that the smaller‐scale studies, based on neuroimaging techniques, reported consistently high‐performance metrics, with three of them reporting AUC values between 0.896 and 0.939 [22, 23, 27]. This contrasts with the larger clinical phenotyping‐based study by Lu et al. [21], which achieved an AUC of 0.744. It is noteworthy that the smaller studies using neuroimaging techniques have high AUCs, while the largest study using clinical phenotyping data has a lower AUC.

##### 3.4.2.2 Triptans

The foundational study by Maas et al. [19] represents the only investigation of triptans, particularly the modeling of the time course of response to sumatriptan. Using a hidden Markov model, this study did not report a classification metric such as AUC, but focused on quantifying the effect of the drug over time and successfully described the progression from “no relief” to “pain free” and estimated the efficacy of the drug (EC_50_).

### 3.5 Evaluation of Model Performance and Generalizability

This section directly addresses research question 4 (*How were the performance and generalizability of these models evaluated*?) by analyzing how the performance and generalizability of the models described above were evaluated in the included studies.

Our analysis, summarized in Table 2, reveals a critical finding regarding the evaluation methodologies used. The vast majority of studies (10 of 12) relied exclusively on internal validation strategies to assess model performance. These methods typically included k‐fold cross‐validation or single train/test splits of data from a single cohort [19, 20, 21, 22, 23, 24, 25, 26, 27]. Rigorous external validation on independent, geographically or temporally separate patient datasets—a crucial step for establishing a model's real‐world generalizability—was exceptionally rare. This gold‐standard approach was attempted in only two of the 12 included studies: Romozzi et al. [28], who validated their model on a separate external cohort, and Wei et al. [29], who performed validation on a public external dataset. This heavy reliance on internal validation methods across the field highlights a significant limitation in the current body of evidence, suggesting that the reported performance metrics may be overly optimistic and may not be maintained in new clinical settings.

### 3.6 Observed Patterns From the Performance‐Cohort Landscape

To provide a holistic overview of the current research landscape, Figure 2 plots predictive performance (AUC, y‐axis) against cohort size (on a logarithmic x‐axis) for each included study. It is critical to note that this is a visual aid intended to illustrate the distribution of the published literature, and not a formal statistical analysis from which conclusions can be drawn. The following observations must be interpreted with extreme caution, due to the heterogeneity of the studies in terms of methodology, patient populations, definition of treatment response, and the high risk of overfitting the model, particularly in smaller cohorts.

#### 3.6.1 An Observed Pattern: The Potential Primacy of Data Modality and Granularity

A visual inspection of the scatterplot does not show a simple linear relationship between reported prediction accuracy and cohort size. Instead, it suggests a possible pattern that should be discussed. For example, in the context of response to NSAIDs, studies by Wei et al. [22, 23, 26, 27, 29] using high‐dimensional neuroimaging data from smaller cohorts (*N* = 64–111) reported AUCs ranging from 0.82 to 0.94. In contrast, the largest NSAID study (*N* = 610), which relied on clinical phenotyping data, reported an AUC of 0.74 [21]. This observation, which should be interpreted with extreme caution, raises the hypothesis that for certain therapeutic questions, the modality and granularity of the input features (e.g., high‐dimensional neuroimaging data versus clinical survey data) may be a more dominant factor in model performance than sample size alone.

#### 3.6.2 Observed Performance for Models of Novel Therapies

Models for preventive CGRP mAbs are a focus of current research, which is reflected in the graph. The three studies for this drug class all reported AUCs above 0.74 [24, 28, 30]. The reported predictive performance ranges from an AUC of 0.746 in one study [28] to 0.825 [30] and 0.980 in the others [24]. Although this difference in performance across the three studies indicates the potential for the development of predictive models for these targeted therapies, it also shows that factors such as the specific model architecture, the granularity of the input data, and the characteristics of the patient cohort are likely to remain critical determinants of a model's success.

#### 3.6.3 Observed Variability in Performance Across and Within Drug Classes

The plot shown in Figure 2 also reveals observable variability in reported model performance both between and within different drug classes. First, the classic oral preventive agents (beta‐blockers, tricyclic antidepressants, topiramate, verapamil, and gabapentin) form a distinct group in the lower right quadrant, with all reported AUCs below 0.70 despite being tested on large cohorts [30]. This observation raises the possibility that predicting response to these broader‐acting agents from routine clinical data may be inherently more challenging, a hypothesis that requires further, dedicated investigation. Second, there is extreme variation in performance within a single class of agents. The models for onabotulinumtoxinA are a clear example. One study, confined to a specific subgroup of 32 patients, reported an AUC of approximately 0.91, though the model failed in a different patient cohort within the same study [25]. Meanwhile, another, larger study based on registry data reported an AUC of 0.58 [30]. This variability suggests that factors beyond drug class alone, such as the specific patient subtype (e.g., high‐frequency episodic vs. chronic migraine), may be critical determinants of model success.

The main research gaps and challenges identified from this body of literature, which directly address research question 5 (*What are the main research gaps and challenges identified in the literature*?), are synthesized and discussed in the Discussion section.

## 4 Discussion

This scoping review has systematically mapped the current landscape of AI/ML applications for predicting response to pharmacologic treatment in migraine. Our review reveals a dynamic but nascent field, one characterized by promising preliminary results but hampered by critical and consistent methodological hurdles that currently prevent any translation into clinical practice. The ultimate goal of these AI/ML applications is to analyze complex patient data to identify “responder phenotypes,” which is considered an important step towards achieving precision medicine in migraine [6]. However, our findings indicate the field is still far from this goal. Our scoping review builds on recent broad surveys of AI in headache medicine, such as the systematic review by Lee et al. [8], which mapped the wide‐ranging applications of these technologies. Although that work provided an essential panoramic overview, the present review offers a distinct and complementary contribution by conducting a focused analysis of the specific challenge of predicting pharmacological treatment response. Our primary aim is not to re‐survey the landscape, but to critically synthesize the existing evidence for this specific application, systematically identify the core methodological barriers—most notably a crisis of generalizability—and propose a concrete research agenda to guide the field towards clinical utility. The studies included in this review are currently dominated by classical supervised learning algorithms, in particular support vector machines and tree‐based ensembles (e.g., random forest). These models have been shown to be highly effective and, in some cases, achieve high reported prediction accuracy (AUCs up to 0.980) when applied to high‐quality data characterized by high dimensionality and detailed features, such as granular clinical phenotyping or high‐dimensional neuroimaging [24, 27]. However, such near‐perfect performance metrics, especially in studies relying solely on internal validation with small cohorts, are a clear warning sign of model overfitting. It is very likely that the stated accuracies are too optimistic and cannot be generalized to new, independent data sets. This strongly suggests that the current bottleneck is not the maturity of the algorithms, but the availability of large and robust data sets suitable for rigorous external validation. The application of deep learning is a recent and significant development. The study by Wei et al. [26] demonstrated the feasibility of using 3D CNNs on raw structural MRI data, which is an important step in applying deep learning architectures to neuroimaging for this task. The more recent study by Chiang et al. [30] is also significant, as it is the first to apply a deep learning model specifically designed for tabular clinical data (TabNet). Taken together, these studies signal a potential shift towards more complex and specialized architectures as the field matures and data availability increases.

Our review shows an observable evolution in the methodologies used over time. The landscape began with a pioneering, theory‐driven approach using a probabilistic Hidden Markov Model to describe pharmacokinetic–pharmacodynamic relationships over time [19]. This was followed by widespread adoption of data‐driven classical ML, which was initially applied to structured clinical data and later to high‐dimensional neuroimaging datasets with considerable success. The most recent wave, represented by studies such as Wei et al. [26] and Chiang et al. [30], marks the emergence of deep learning architectures. Although this evolution from theory‐driven to data‐driven to algorithm‐driven models reflects the increasing maturity of the field, our central finding is that this has not been accompanied by a parallel evolution in methodological rigor. Regardless of the algorithm's complexity, the fundamental limitations identified in our review—most notably the lack of external validation—remain a constant and critical barrier to progress.

An interesting pattern that emerges from the literature is an apparent performance gap between models for novel, targeted therapies and those for older, classical agents. Models for CGRP‐mAbs and NSAIDs (especially with neuroimaging) have high reported AUCs, while the models for classical oral preventive agents have lower reported AUCs. However, it would be inappropriate to conclude from our review that this is an intrinsic property of the drugs themselves. It is highly plausible that this apparent gap is a direct reflection of a data quality bias, as noted in the included studies themselves. Models for older drugs are often based on less detailed registry data, whereas models for newer agents and NSAIDs have been developed using dedicated, high‐granularity research datasets. Therefore, a key finding of our review is the need for studies that apply the same high‐dimensional phenotyping and neuroimaging methods to older preventive agents. Such work is required to determine if their lower reported predictability is an intrinsic drug property or, more likely, an artifact of the less‐detailed data used for modeling to date.

Although the potential of AI/ML in this field is evident from the included studies, the main finding of our review is not that it is promising, but that there are significant gaps in the current evidence. A related issue that exacerbates the “data bottleneck” is the evidence of overlapping patient cohorts in the literature. Our review identified five distinct studies from the same institution that appear to be based on analyses of the same, or largely overlapping, patient datasets [22, 23, 26, 27, 29]. This practice, often referred to as “salami slicing”, is a critical problem for the field, as it can falsely inflate the evidence base by presenting analyses from a single cohort as independent sources of evidence. Although we included these studies in our scoping review to fully capture the analytic techniques used, this finding highlights the limited diversity of data sets currently available. Furthermore, a key finding is the almost complete absence of models that integrate genomic, proteomic, or other “omics” data, despite clear evidence of their potential to subdivide migraine and predict onset [11, 31, 32]. Equally notable is the lack of studies using data from wearable devices such as smartwatches or sleep trackers that could provide continuous, objective, and real‐world data on physiological parameters, sleep patterns, and activity levels [14, 33]. This lack of multimodal data integration, a direct result of our systematic search, represents a significant blind spot for the ambitions of precision medicine and a major gap in the current literature. The future vision for the field must include multimodal integrations and envisions “digital twins” of patients that combine clinical, genomic, and real‐time wearable data to create a holistic prediction framework [14]. Furthermore, the focus of research is unbalanced. Considerable effort is directed towards newer, high‐cost preventive therapies such as CGRP‐mAbs and onabotulinumtoxinA, while their predictability varies from study to study. In stark contrast, models for classical oral preventive agents showed only modest performance [30]. Although this review includes an early pharmacokinetic model for sumatriptan [19], no AI/ML prediction models were identified for other drugs in the triptan class. Conspicuous gaps also remain for other widely used acute treatments such as small molecule CGRP receptor antagonists and serotonin 5‐HT1F receptor agonists [34]. This imbalance suggests that research priorities are driven by therapeutic novelty rather than clinical breadth, leaving clinicians without predictive tools for many common treatment decisions. This is particularly unfortunate as the clinical history may contain valuable predictive information. For example, in a recent systematic review and meta‐analysis of CGRP‐mAbs, a good prior response to triptans was identified as a strong and consistent predictor of a positive outcome, suggesting a common underlying CGRP‐dependent mechanism that could be used for patient selection [35].

Perhaps the most important finding to emerge from our systematic review of the 12 included studies is the confirmation of a “crisis of generalizability” [36]. Our analysis of the validation methods used (Table 2) shows that the reported high performance metrics are based almost exclusively on internal validation, which carries a high risk of bias and overfitting. Rigorous external validation on independent, geographically or temporally separated datasets is exceptionally rare and was only attempted in two of the 12 included studies [28, 29]. In the two cases where external validation was performed, results were mixed: Wei et al. [29] reported a predictable drop in performance, with ROCAUC decreasing from 0.711 on the internal test set to 0.631 on an external public dataset. In contrast, Romozzi et al. [28] observed stable performance, with the AUC remaining consistent between the internal (0.76) and external (0.78) cohorts, a result that highlights the complexity of assessing model generalizability. This finding is consistent with a recent systematic review and meta‐analysis by Chen et al. [37], which analyzed a partially overlapping set of studies. Their quantitative synthesis, combining predictive models for both pharmacological and nonpharmacological treatments such as acupuncture and neurostimulation, yielded a promising pooled AUC of 0.86. However, the authors strongly cautioned against this interpretation, pointing out the exceptionally large heterogeneity between studies (*I* ^2^ = 90.8%) due to the different interventions. Most critically, they found a consistently high risk of bias in the “Analysis” section of the PROBAST assessment, primarily due to the lack of external validation and inadequate sample sizes. This confirms that, despite its promise, the field has not yet adopted the rigorous validation standards that are becoming commonplace in other neurological and psychiatric disciplines. As such, the field urgently needs to adopt robust validation approaches from related disciplines such as epilepsy and psychiatry, where this challenge is being actively addressed [13, 38].

The path from computational promise to clinical reality requires a radical shift in research priorities. Our findings are a clear call to action to overcome the “data bottleneck” by fostering large‐scale, multi‐center collaborations to generate the diverse, robust datasets that are currently lacking [32]. This is not only a logistical challenge, but also a deeply ethical one, as models trained on unrepresentative data risk perpetuating or exacerbating existing inequalities in health care [39]. To resolve the critical crisis of generalizability, journals and funders must require external validation as a non‐negotiable condition of publication. To solve the “black box” problem, which is particularly evident in deep learning models such as that of Wei et al. [26], the integration of explainable AI techniques (XAI) is a necessity to build trust and ensure clinical accountability [40]. The ultimate goal is to develop systems that can predict differential response to treatment and help clinicians select the best option for an individual patient—a goal already being pursued in depression using large‐scale Electronic Health Record (EHR data) [41]. These powerful tools must complement clinical care, not hinder it [42]. Once the research community has overcome the fundamental challenges of validation and generalizability, a critical future direction will be to synthesize these findings into clinically oriented publications and decision aids that provide clear, actionable “pearls” for daily practice. In doing so, they must be based on sound ethical frameworks that encompass the entire lifecycle of AI‐driven medical software [43]. To systematically address these critical barriers, we have summarized our findings in a structured agenda for future research (Table 5), which provides a concrete roadmap for future investigations.

**TABLE 5:** A proposal for a future research agenda to advance AI/ML in the pharmacological treatment of migraine.

Research priority area	Key challenge(s) identified	Recommended actions and future directions
1. Ensuring model reliability and generalizability	“Crisis of generalizability”: Over‐reliance on internal validation leads to overly optimistic and non‐transferable models. External validation is exceptionally rare	Action: Mandate rigorous external validation on independent datasets as a prerequisite for publication and funding. Future direction: Progress to prospective validation studies to test model performance and clinical utility in real‐world settings
2. Overcoming data scarcity and bias	“Data Bottleneck”: Most research relies on small, single‐center, and often ethnically homogeneous datasets. Evidence of overlapping cohorts (“salami slicing”) was also identified	Action: Foster large‐scale, multicenter collaborations and international consortia to create diverse, robust datasets. Future direction: Establish CDEs and standardized data collection protocols to ensure interoperability and facilitate meta‐analyses
3. Expanding data modalities for precision	Incomplete biological picture: A complete absence of models incorporating genomic, proteomic, or other “omic” data (e.g., from serum, CSF)	Action: Design studies to explicitly test the predictive value of biomarkers and genetic data in addition to clinical and imaging features. Future direction: Develop unified, multimodal models that integrate diverse data streams to create holistic “digital twins” of patients
4. Broadening the therapeutic and clinical scope	Unbalanced research focus: A major gap in predictive models for widely used acute treatments (especially gepants and ditans) and modest performance for classical preventives	Action: Prioritize the development of models for common acute medications, including modern therapies like gepants. Future direction: Investigate novel, more granular features to improve the predictive accuracy for older, broader‐acting preventive drugs
5. Building clinical trust and interpretability	The “Black Box” problem: The opacity of complex models, especially deep learning architectures, undermines clinical confidence and adoption	Action: Systematically integrate XAI techniques into the model development and reporting process. For example: SHAP to fairly attribute the prediction outcome to each feature's contribution. LIME to explain a single prediction using a simple, local substitute model. Future direction: Use insights from predictive features to define new, data‐driven “responder phenotypes,” enhancing the pathophysiological understanding of migraine

Abbreviations: AI/ML: artificial intelligence/machine learning; CDEs: common data elements; CSF: cerebrospinal fluid; LIME: local interpretable model‐agnostic explanations; SHAP: SHapley Additive exPlanations; XAI: explainable AI.

It is also important to recognize the limitations of this review itself. One important limitation is the available evidence base. Our systematic search found only 12 studies, a number that reflects the early stage of development of AI/ML applications for predicting response to migraine treatment. This lack of published peer‐reviewed articles emphasizes the value of this review in consolidating the current sparse evidence and identifying clear directions for future research. Second, like all literature reviews, this work is susceptible to publication bias. Third, in line with the aims of a scoping review, we have not formally assessed risk of bias for the included studies, which means that the performance measures they report should be interpreted with caution. Finally, the rapid pace of innovation means that this review is a snapshot in time. However, these limitations do not detract from the main aim of the review, which is to present the current state of play and identify key challenges within the published evidence base.

## 5 Conclusions

In summary, our scoping review of the available evidence shows that the application of AI/ML to predict response to pharmacological treatment of migraine is a nascent field, not yet a promising one in practice. Although our review found that some studies report high predictive accuracy with classical models such as tree‐based ensembles, our central finding is that this apparent success is undermined by critical and pervasive methodological weaknesses that currently prevent any clinical application of these models. The most important problem, identified consistently across the reviewed literature, is the “crisis of generalizability” due to an almost invariable reliance on internal validation, resulting in performance metrics that are likely to be overly optimistic and not translatable to real‐world clinical practice. This is compounded by a “data bottleneck” due to reliance on small, single‐site studies and a notable lack of predictive models for many widely used acute treatments. To bridge the gap between research and clinical practice, as our findings make clear, future efforts must prioritize the creation of large, diverse, multicenter datasets, establish external validation as a mandatory standard, and integrate XAI to build clinical confidence. Overcoming these fundamental challenges is essential to begin to unlock the true potential of AI to personalize migraine treatment and significantly improve patient outcomes.

## Author Contributions

**Martina Giacon:** writing – original draft, investigation, methodology, formal analysis, data curation, visualization. **Salvatore Terrazzino:** conceptualization, supervision, data curation, writing – original draft, writing – review and editing, investigation, methodology, visualization, formal analysis, validation.

## Funding

The authors have nothing to report.

## Conflicts of Interest

The authors declare no conflicts of interest.

## Supporting information


**Table S1:** Preferred Reporting Items for Systematic reviews and Meta‐Analyses extension for Scoping Reviews (PRISMA‐ScR) Checklist.
**Table S2:** List of the search strings applied to each database.
**Table S3:** Detailed predictive models and features from Chiang et al. 30.

## Data Availability

Data sharing not applicable to this article as no datasets were generated or analysed during the current study.
